# Cross modality learning of cell painting and transcriptomics data improves mechanism of action clustering and bioactivity modelling

**DOI:** 10.1038/s41598-025-05914-0

**Published:** 2025-07-02

**Authors:** Son V. Ha, Steffen Jaensch, Maciej M. Kańduła, Dorota Herman, Paul Czodrowski, Hugo Ceulemans

**Affiliations:** 1 Johnson & Johnson, Beerse, Belgium; 2 Johnson & Johnson, Vienna, Austria; 3 Department of Chemistry, Johannes Gutenberg University Mainz, Mainz, Germany

**Keywords:** Cell biology, Computational biology and bioinformatics, Drug discovery

## Abstract

In drug discovery, different data modalities (chemical structure, cell biology, quantum mechanics, etc.) are abundant, and their integration can help with understanding aspects of chemistry, biology, and their interactions. Within cell biology, cell painting (CP) and transcriptomics RNA-Seq (TX) screens are powerful tools in early drug discovery, as they are complementary views of the biological effect of compounds on a population of cells post-treatment. While multimodal learning of chemical structure-cell painting, or different omics data has been experimented; a cell painting-bulk transcriptomics multimodal model is still unexplored. In this work, we benchmark two representation learning methods: contrastive learning and bimodal autoencoder. We use the setting of cross modality learning where representation learning is performed with two modalities (CP and TX), but only cell painting is available for new compounds embeddings generation and downstream task. This is because for new compounds, we would only have CP data and not TX, due to high data generation cost of the RNA-Seq screen. We show that in the absence of TX features for new compounds, using learned embeddings like those obtained from Constrastive Learning enhances performance of CP features on tasks where TX features excels but CP features does not. Additionally, we observed that learned representation improves cluster quality for clustering of CP replicates and different mechanisms of action (MoA), as well as improves performance on several subsets of bioactivity tasks grouped by protein target families.

**Supplementary Information:**

The online version contains supplementary material available at 10.1038/s41598-025-05914-0.

## Introduction

Self-supervised representation learning is an important aspect of machine learning^1–4^, leveraging abundant unlabelled data to help models learn embeddings that capture underlying structures and patterns. These embeddings are beneficial for a variety of downstream tasks where labels for supervised learning are rare. In drug discovery, learning useful representations from chemical^5,6^ or biological data^7,8^ is similarly useful. This is because the representations can not only improve performance in downstream modelling tasks with few labelled data, but also enhance our understanding of chemistry, biology, and their interactions.

In small molecule drug discovery, high content screens such as cell painting^9^ or RNA-Seq^10^ are often used to quantify the changes in the biological system induced by a perturbation (e.g., after application of a treatment). The results are morphological profiles (CP data, short for ‘Cell Painting’) from cell painting or gene expression profiles (TX data, short for ‘Transcriptomics’) from RNASeq for compounds, which can be further analysed to enhance our understanding of the biological effect of different drugs. Application of CP data includes modelling of small molecules activity in different biological assays^11–13^, and determining mechanism of actions of compounds^14–16^. Application of TX data includes annotating cell types^17^, identifying differentially expressed genes to study their function^18,19^, and discovering potential novel biomarkers^20^.

Multimodal learning is a subfield in machine learning that aims to process and integrate data from multiple modalities, originally with the goal to mimic the way human combine information from different senses (sight, sound, touch, etc.). Multimodal learning has made significant progress in recent years^21^, from language-vision models^22,23^, video captioning^24^, autonomous driving^25^, to biomedical AI^26^. One of the principles underlying these rapid developments is the increasing adoption of self-supervised learning (SSL) methods^27^, which allowed for models to be trained at an unprecedented scale. Instead of relying on expensive human annotated labels, SSL utilizes supervision from abundantly available unannotated data, and is then finetuned to a variety of downstream tasks of interest with little data. This technology has potential to be extremely beneficial in drug discovery, as there is a large diversity of data sources such as chemical structure, cell images, omics, quantum chemistry, etc. There has been research on multimodal learning of chemical structure-cell painting^8,28–30^, and between single-cell RNA-seq and chromatin images^7^. However, to the best of our knowledge multimodal learning of cell painting (CP) data and bulk transcriptomics (TX) data are still underexplored.

In this work, we study cross modality learning^31^ of CP and TX data: a multimodal representation learning setting where we try to learn better single modality representations from CP data given unlabelled data from both CP and TX. The reason for this is that for new compounds, we would most likely only have CP data and not TX, because generating TX data is much more costly than CP. Ye et al.^32^ make an estimation of a scalable RNA-Seq setup, with $2–4 per sample. In our experience, generating RNA-Seq (TX) data costs ~ $6–10 per well. This makes it substantially more expensive than images (CP), which we typically generate at ~ $0.50–$1 per well, depending on the cell line. Moreover, RNA-Seq experiments are substantially harder to scale to > 100 k compounds as it is more labor-intensive to prepare the samples for RNA-Seq and generate the data.

Because of that, a practical representation learning algorithm must be able to learn representation on both modalities, but only need CP data for embeddings generation. More specifically, we benchmark two cross modality representation learning methods: contrastive learning (CL) and bimodal autoencoder (BAE), on a variety of unsupervised and supervised downstream tasks. We show that learned representation improves cluster quality for clustering of CP replicates and different mechanisms of action (MoA), with CL embeddings yielding the best results. In the supervised bioactivity multitask classification, we demonstrate that CL embeddings achieves higher mean AUROC and RIPtoP-AUPRC compared to CP features across a range of bioactivity tasks. Additionally, we provide a more detailed comparison of features performance on bioactivity tasks grouped by protein target families. Finally, we show that in the absence of TX features for new compounds, using learned embeddings enhances performance of CP features on tasks where TX features excels but CP features does not.

## Related works

### Representation learning of cell painting data

Learning useful representations from Cell Painting data, either in image form or as extracted features (using CellProfiler^33^ or PerkinElmer (Waltham, MA) Acapella [https://content.perkinelmer.com/lab-products-and-services/product-support.html]), is useful for leveraging the rich amount of information that Cell Painting provides. Efforts have been made to learn representations unimodally from (or pre-train on) from cell images using self-supervised methods like DINO, SimCLR and MAE^34,35^.

Additionally, multimodal representation learning for molecules has utilized Cell Painting data as the second modality^8,28–30^ to introduce biological information into molecular structure-based representations. The aim is not only to enhance performance in modelling tasks such as Quantitative Structure–Activity Relationship (QSAR) but also to establish a link between chemical structure and biological phenotypes. This can be observed in the downstream tasks introduced by the aforementioned works, such as the mutual retrieval of molecules and their corresponding images.

### Representation learning of transcriptomics data

To the best of our knowledge, there has not been a public bulk transcriptome dataset at the same scale as the Janssen dataset, hence the lack of literature. The vast majority of literature on representation learning using gene expression data has been using single-cell transcriptome, due to the availability of public data. These methods range from variational autoencoder^36^, to transformer-based models^37–39^ based on BERT^4^ and GPT^40^. These models have shown that self-supervised pretraining on a large single-cell transcriptome corpus (30 to 50 million cells equivalent to 600 billion to 1 trillion tokens) as an effective strategy to boost modelling accuracy in a variety of downstream tasks. Lastly, Yang et al. used an autoencoder architecture to translate between single-cell RNA-seq and chromatin images^7^.

## Methods

### Data generation

#### Cell painting image acquisition and processing

Images of chemically perturbed cells were acquired using Cell Painting, a high-content image-based assay for morphological profiling. In short, U2OS cells purchased from ATCC (HTB-96) were seeded in 1536 well plates and allowed to attach for 24 h. Compounds were diluted in DMSO to the final concentration at 10µM. We label different cell components or organelles using the same fluorescent dyes as described in Bray et al.^9^. Images of the five fluorescence channels were acquired with a Yokogawa (Tokyo, Japan) CellVoyager 8000 confocal high-content imaging reader. Then the PerkinElmer (Waltham, MA) Acapella version 4.1.3 (https://content.perkinelmer.com/lab-products-and-services/product-support.html) image analysis software was used to extract around 800 morphological features from individual cells, such as staining intensity, texture, shape and spatial correlations. After that, quality control, well-level aggregation and normalization against the DMSO control on every plate are performed. In the end, we obtain a vector of Z-scores for each compound, which we will call a Cell Painting profile. More details about can be found in^12^ under ‘Experimental Procedure/Data Preparation/Cell Painting Images and Image Preprocessing’. In addition, details about the stained cellular components, types of dyes and others can be found in Table 1 of Bray et al.^9^.

**Table 1:** Performances of each features type for 47 bioactivity classification tasks that TX performs well (AUROC > 0.7) and CP does not perform well (AUROC < 0.7).

Features Type	Mean AUROC	Mean RIPtoP-AUPRC	# (AUROC > 0.7)	# (AUROC > 0.8)
CP	0.641 ± 0.04	0.359 ± 0.11	0	0
CL Emb	0.671 ± 0.06	0.407 ± 0.15	14	1
BAE Emb	0.656 ± 0.06	0.373 ± 0.12	13	0
TX	0.736 ± 0.03	0.468 ± 0.09	47	1

Mean metrics ± standard deviation metrics for the mean AUROC and mean RIPtoP-AUPRC columns. #(AUROC > 0.7) denotes number of tasks that achieves AUROC > 0.7.

#### Transcriptomics data generation with bulk RNA-Seq

U2OS cells were seeded at a density of 1600 cells/well in 384 plates on day 1.24 h later, on day 2, compound treat was performed with the compounds of interest + DMSO (vehicle) using the Echo Liquid Handler. Again, 24 h later, on day 3, medium was removed from the cells using the BlueWasher (Lightspin) followed by addition of 20 µl of Cells-To-Signal lysis buffer (diluted 1/3 in PBS) per well with Multidrop. Plates were kept for 10 min at room temperature to get proper lysis and eventually stored at − 80 °C. After lysis, the actual HT-RNAseq protocol started. In the first step of the protocol, double-stranded complementary DNA (cDNA) was synthesized from each well with each sample being uniquely barcoded. After that, all wells from a 384 well plate were pooled together to one single tube followed by a Kapa HyperPlus Library preparation using Illumina-compatible adapters following the manufacturer’s introductions. Afterwards, libraries were sequenced on an Illumina NovaSeq 6000 S4 instrument to an average depth of 1M reads per well.

Sequencing reads were aligned using STAR solo v2.7.6a^41^ with default parameters, except for: (outFilterMultimapNmax: 1, soloCBstart: 1, soloCBlen: 10, soloUMIstart: 11, and soloUMIlen: 10), towards the human reference genome GRCh38.102 extended with a list of 92 ERCC spike-ins. The resulting UMI counts were analysed using R. After filtering for Havana and Ensembl genes, variance stabilizing transformation was applied to all samples within a plate, followed by a library size correction, taking compound and dose treatment into account, using DESeq2^42^ and limma^43^ R packages. Relative data versus vehicle was then calculated for each treatment within a plate such as robust adjusted Z-scores used in our work: (Sample–Median(DMSO))/StandardDeviation (DMSO)).

### Evaluation methodology

**Fig. 1:**
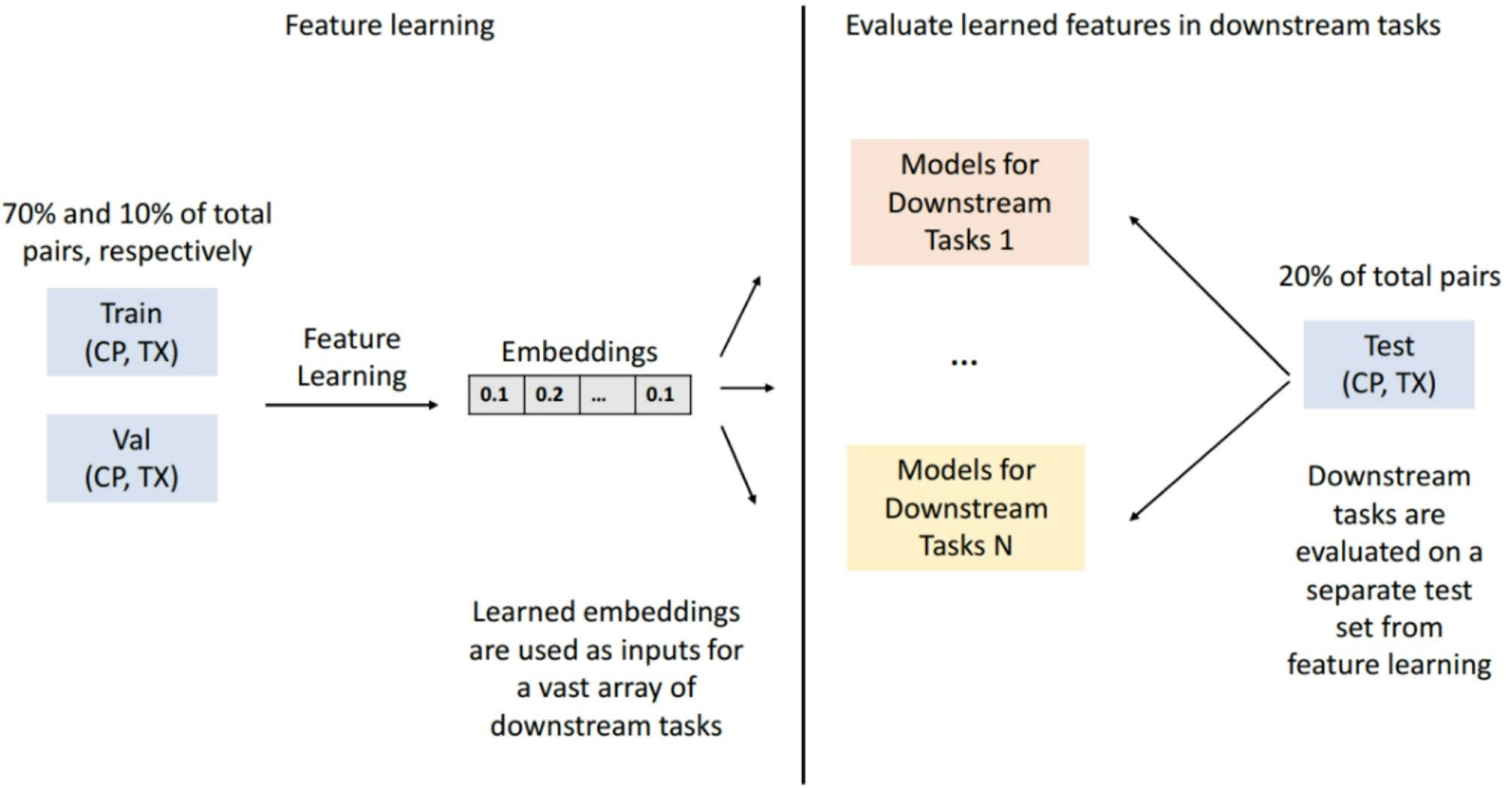
Evaluation methodology. We split the dataset into train, validation and test set with the ratio 70/10/20. We perform features learning on the train and validation tasks and evaluate the learned embeddings against the original CP and TX features on the test set.

In our dataset, each unique compound has one TX profile and several CP profiles due to replicate measurements, with the number of replicates varying across compounds. We group these into (CP–TX) pairs, resulting in just over 200 k pairs for approximately 100k compounds. We then split the data into training, validation, and test sets (70/10/20) based on the Murcko Scaffolds^44^ of the compounds, ensuring that structurally similar compounds are in the same set. The aim is to make training harder and more robust, because compounds structurally similar to the training set are not included in validation or testing. To enable a fair comparison of each features learning algorithm, we perform features learning on the training and validation sets and evaluate their performances across various downstream tasks using the hold-out test set (Fig. 1).

### Contrastive pretraining

The first features learning algorithm we benchmark is Contrastive learning (CL): a method that involves optimizing the InfoNCE objective^2,3^ across two learned embeddings from two separate encoders. For each batch with size N, the InfoNCE is as follows:

$$L_{{{\text{InfoNCE}}}} = - \frac{1}{N}\mathop \sum \limits_{i = 1}^{N} \ln \frac{{\exp \left( {{\text{sim}}\left( {x_{i} ,z_{i} } \right)/{\uptau }} \right)}}{{\mathop \sum \nolimits_{j = 1}^{N} \exp \left( {{\text{sim}}\left( {x_{i} ,z_{j} } \right)/{\uptau }} \right)}} - \frac{1}{N}\mathop \sum \limits_{i = 1}^{N} \ln \frac{{\exp \left( {{\text{sim}}\left( {x_{i} ,z_{i} } \right)/{\uptau }} \right)}}{{\mathop \sum \nolimits_{j = 1}^{N} \exp \left( {{\text{sim}}\left( {x_{j} ,z_{i} } \right)/{\uptau }} \right)}}$$

where ${\text{sim}}\left( {x_{i} ,z_{i} } \right) = x_{i}^{T} z_{i} /\left| {\left| {x_{i} } \right|} \right|\left| {\left| {z_{i} } \right|} \right|$ is the cosine similarity between the output of CP encoder $x_{i}$ and $z_{i}$. ${\uptau }$ is a temperature parameter which controls how concentrated the features are in the representation space^45^. Smaller $\tau$ will make widely separated representations irrelevant, which heavily degenerate the performance.

**Fig. 2:**
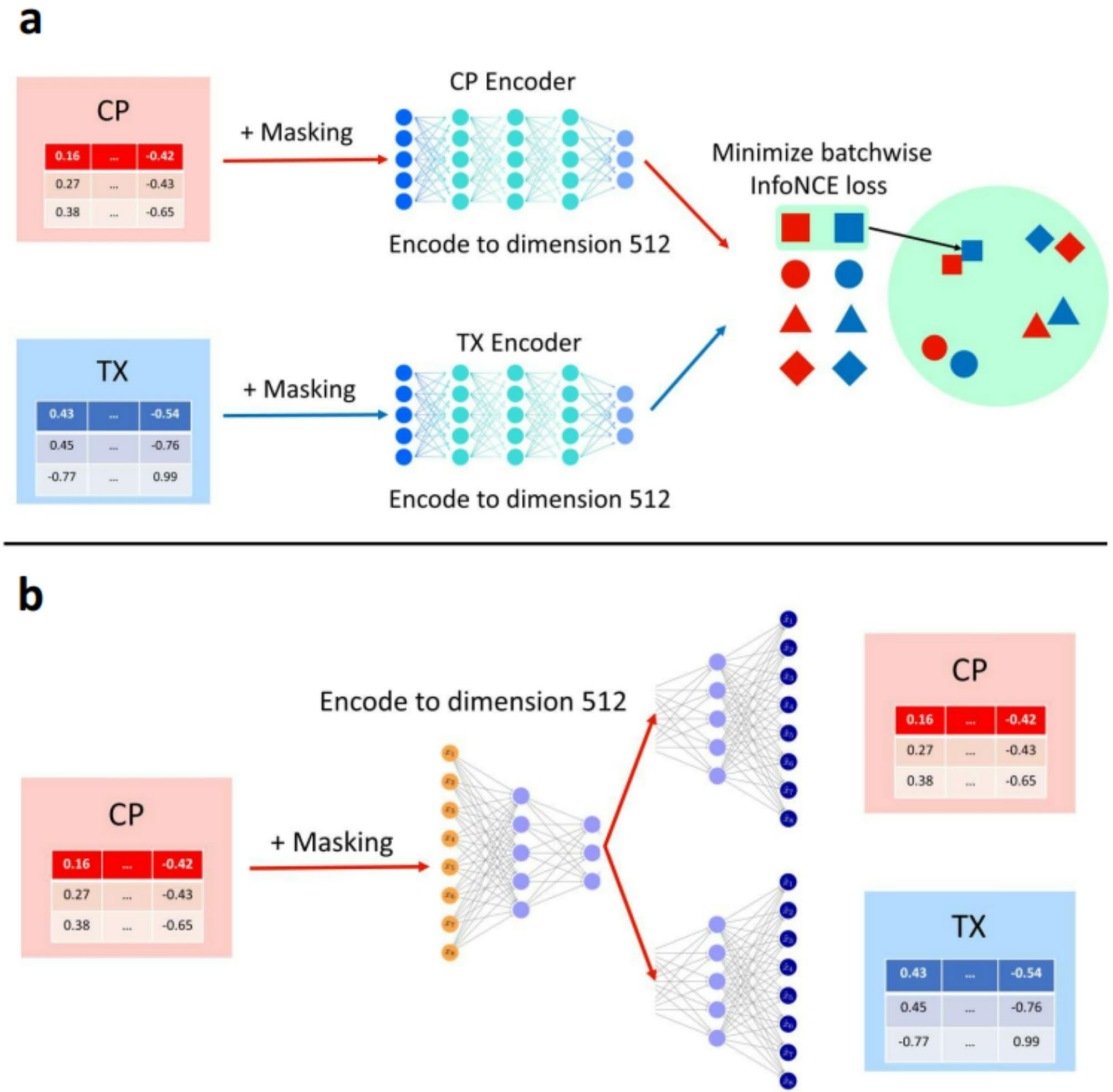
Schema of 2 different pretraining methods. (**a**) Contrastive learning. (**b**) Bimodal autoencoder. For both pretraining methods data are augmented with 10% masking before being encoded. Contrastive learning pretraining aims to minimize the InfoNCE loss between pairs of the same compounds and pairs of different compounds in a batch. Bimodal autoencoder pretraining aims to minimize the average of mean square error of each reconstruction.

InfoNCE maximizes the similarity between the correct pairs (CP and TX profile of the same compound) and minimizes the similarity between the other random (CP, TX) pairs in the latent space. In our cross modality setting, we use both CP and TX data to pre-train the two CP and TX encoders (Fig. 2). When generating embeddings for new compounds that do not have TX data, we would only need the CP encoder.

Our contrastive learning framework (Fig. 2) includes a CP encoder and a TX encoder, both of which are fully connected neural networks (MLPs). The CP encoder has hidden layers of sizes [1024, 1024, 1024], while the TX encoder has hidden layers of sizes [4096, 4096, 4096]. Both encoders have output size 512. Since data augmentation is often useful in contrastive learning^2,46^, we apply an augmentation to the CP and TX data before encoding: randomly masking 10% of the input features. In addition, we use a linear projection to map from each encoder’s output (dimension 512) to a lower dimension 256, similar to the CLIP model^1^. For embeddings generation, the projection head is discarded, and we only use the MLP encoder. We tested 3 hyperparameter configurations for [CP_encoder_hidden_layers_size, TX_encoder_hidden_layers_size]: [512, 4096], [1024, 4096], [1024, 8192]. This hyperparameter search gave satisfactory results already, especially considering the high computational cost of each run (each pretraining run takes between 1 and 3 weeks to complete).

### Bimodal autoencoder pretraining

Bimodal autoencoder (BAE)^31^ involves one encoder for CP data, and two decoders for CP and TX data. The output of the CP encoder is the learned embeddings (also known as ‘latent vector’ in autoencoder literature), which can be generated with the CP encoder using only CP data for new compounds. The model is trained to reconstruct both modalities when given only CP data, minimizing the average mean squared error (MSE) of the two reconstructions:

$$L_{{\text{MSE - BAE}}} = \frac{1}{2}\mathop \sum \limits_{i = 1}^{N} \left( {u^{\prime}_{i} - u_{i} } \right)^{2} + \frac{1}{2}\mathop \sum \limits_{i = 1}^{N} \left( {v^{\prime}_{i} - v_{i} } \right)^{2}$$

where $u_{i}$ and $v_{i}$ are original CP and TX data, and $u^{\prime}_{i}$ and $v^{\prime}_{i}$ are CP and TX reconstructions (output of the CP decoder and TX decoder).

The CP encoder, CP decoder and TX decoder have hidden layers [1024, 512, 512], [512, 512, 1024] and [1024, 2048, 4096] (Fig. 2). The size of the embeddings is 512. We also apply an augmentation that randomly mask 10% of the input features. This can be thought of as a masked autoencoder. More details about hyperparameters for both pretraining methods can be found in the Supplementary Information. We tested 3 hyperparameter configurations for [embedding_size]: [128], [256], [512] and chose 512 for the same reason mentioned in the Contrastive Learning section.

### Metrics

#### Binary classification

To assess model performance in binary classification downstream tasks, we use Area under the ROC Curve (AUROC) and Precision-Recall curve (AUPRC). We apply Relative Improvement of Proximity to Perfection (RIPtoP)^47^ correction to AUPRC to ’rescale’ AUPRC so that for every assay, 1 is the perfect model and 0 is the random baseline. The formula for RIPtoP correction is:

$$RIPtoP\left( {AUPRC} \right) = \frac{AUPRC - BASELINE}{{1 - BASELINE}}$$

#### Clustering quality

Assessing which features type produces better cluster quality in unsupervised downstream tasks is typically done with human judges. They examine a 2-D plot obtained from a dimension reduction algorithm (e.g., t-SNE) for expected clusters. This approach, however, cannot be practically applied when there are a lot of data points and clusters which can be unclear to the human eye. Hence, we also use a systematic metric for clustering quality called kNN accuracy (k-nearest neighbour accuracy)^8^.

The intuition behind this metric is, if one features type produced higher-quality cluster than another, fitting a k-nearest neighbour classifier on that features type (with the label being the true cluster label) would yield higher accuracy. For our implementation, we use the function KNeighborsClassifier with default setting (neighbours = 5) from scikit-learn^48^ to produce cluster classifications. Then accuracy is calculated between them and the true cluster labels.

## Results

### Bioactivity multitask classification—tasks that TX performs well but CP does not

We explored the cross-modality learning setting of generating TX data via RNA-Seq compared to Cell Painting data. Consequently, new compounds were more likely to have only cell painting data, and could no longer take advantage of well-performing transcriptomics models. As a result, in this section, we compared the different features types in the context of supervised bioactivity multitask classification^11,12^, which had applications in early drug discovery including virtual screening, hit triaging, and prioritizing hits for experimental follow-up. In particular, this analysis focused on tasks where TX features exceled but CP features did not, aiming to assess whether the learned embeddings could enhance CP features performance in these tasks.

This multitask approach involved training a single model across multiple bioactivity tasks simultaneously, leveraging task correlations to enhance performance. All modelling tasks were binary classification tasks, obtained from binarizing bioactivity assays which originally measured potency (pIC50) of a compound. We trained a multitask model on the compounds in the train and validation set, and evaluated on compounds in the test set. The bioactivity label matrix contained thousands of binarized bioactivity tasks. For model training we only selected tasks that have at least 25 positive, 25 negatives, and at least 100 overall datapoints.

The model architecture was a 3-layer MLP whose hyperparameters were: (hidden layer size = 256, optimizer = Adam, learning rate scheduler: Cosine Annealing Warm Restarts which resets every 10 epochs learning rate = 1e−4, weight decay = 1e−5, batch size = 128, epochs = 100, dropout probability = 0.3). For each features type (CP, TX, CL Emb, BAE Emb), we trained the multitask bioactivity model using the exact same setting to ensure the only variable was the features types themselves. For evaluation, we reported results on the subset of assays that satisfied the following properties:

- Had at least 25 positive and 25 negative in the test set, to avoid too few positives or negatives,
- TX features achieved an AUROC > 0.7 and CP features had an AUROC < 0.7.

This process results in 47 tasks.

When using TX features, the mean AUROC and RIPtoP-AUPRC were 0.736 and 0.468, respectively (Table 1). On the other hand, the mean scores for CP features were lower, at 0.641 ± 0.04 and 0.359 ± 0.11. Both CL and BAE embeddings improved upon the performance of CP features. CL embeddings achieved the higher mean scores between the two (0.671 ± 0.06 and 0.407 ± 0.15), with statistically significant improvements in both metrics at the 0.05 level, according to the Wilcoxon signed-rank test (Supplementary Table 13 and Supplementary Table 14). BAE embeddings showed slightly lower mean scores (0.656 ± 0.06 and 0.373 ± 0.12), with only the AUROC improvement being statistically significant, even though the mean RIPtoP-AUPRC was still higher than that of CP features. Furthermore, the learned embeddings improved 14 tasks for CL embeddings and 13 tasks for BAE embedding, out of the 47 tasks, to an AUROC > 0.7.

### CP replicates clustering

We perform additional unsupervised and supervised analysis in Section. “CP replicates clustering, “Mechanisms of action clustering”, “Bioactivity multitask classification—wider range of tasks”.

CP replicates clustering is an unsupervised denoising task that investigates how well each features type clusters CP replicates. As mentioned in the previous section, each compound has several CP replicates. As a result, when we performed unsupervised analysis, CP replicates of the same compound were assumed to lie in the same cluster, and CP replicates of different compounds were assumed to lie in different clusters. We calculated kNN accuracy for compounds in the test set for each features type, with the true cluster labels being test set compounds (Table 2). CL embeddings achieved the highest kNN accuracy at 0.805, drastically improving on the original CP feature, whose kNN accuracy is 0.416. BAE embeddings achieved 0.428, a minor improvement to that of CP feature.

**Table 2:** Clustering quality of each features type, as measured by kNN accuracy in two unsupervised downstream tasks.

Features type	kNN accuracy CP replicates	kNN accuracy MoA
CP	0.416	0.784
CL Emb	0.805	0.952
BAE Emb	0.428	0.784

Contrastive learning embeddings demonstrates superior clustering of CP replicates and different MoA over the original CP feature. BAE embeddings only improves kNN accuracy by a small amount over CP feature.

**Fig. 3:**
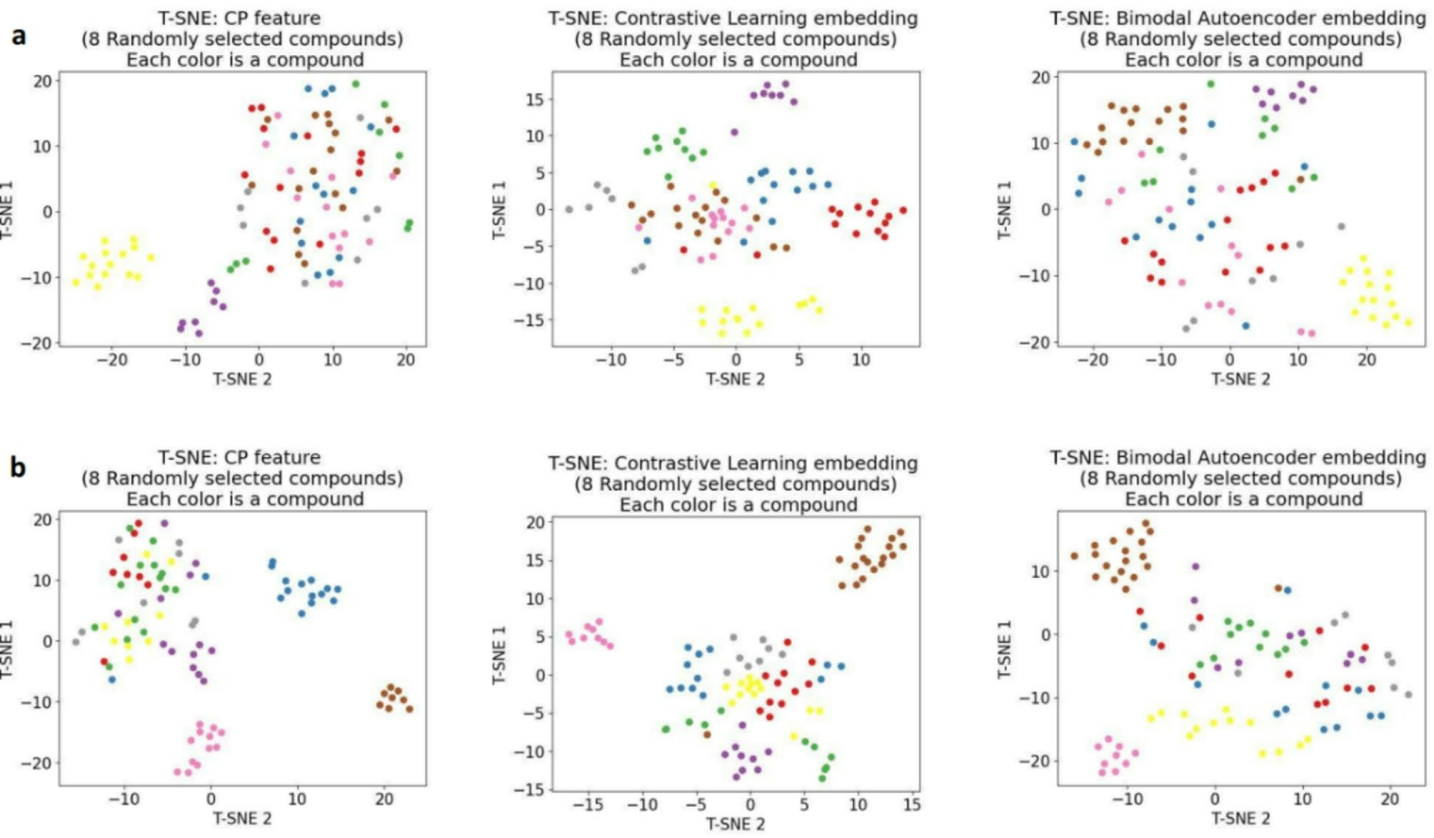
T-SNE plots demonstrating replicate clustering ability of CP embeddings for (**a**) 8 randomly selected compounds. (**b**) other 8 randomly selected compounds. It can be observed that the embeddings, especially CL, improves clustering ability of CP replicates over the original CP feature.

For visualization, we also produced t-SNE plots for eight randomly selected compounds from the test set (Fig. 3a and b) using the function sklearn.manifold.TSNE from scikit-learn^48^ with parameters (learning rate = ’auto’, init = ’random’, perplexity = 10). Each colour corresponded to one compound in the plots. We did not plot the entire test set because the large number of compounds meant there are thousands of colours in the plot, making clusters indiscernible. Overall, it could be observed that the t-SNE plots showed a consistent trend with the kNN accuracy table, as CL embeddings displayed superior clustering ability over the other features types. For example, in Fig. 3a, only the yellow and purple clusters were visible for CP features and BAE embedding. In contrast, CP replicates of the same compounds were much more clearly clustered when using CL embedding.

### Mechanisms of action clustering

Another unsupervised task of interest is the clustering of different Mechanisms of Action (MoA)^28,49^, which gives insight into which compounds share the same MoA. Similar to the first unsupervised experiment, here we calculate the kNN accuracy for each features type, in addition to visually inspecting a t-SNE plot.

We selected 9 mechanisms of action with the largest number of compounds in the test set. KNN accuracy was calculated for compounds in the test set for each features type, with the true cluster labels being the 9 MoA classes. CL embeddings again achieved the highest kNN accuracy at 0.952 compared to CP features and BAE embedding, both at 0.784 (Table 2).

**Fig. 4:**
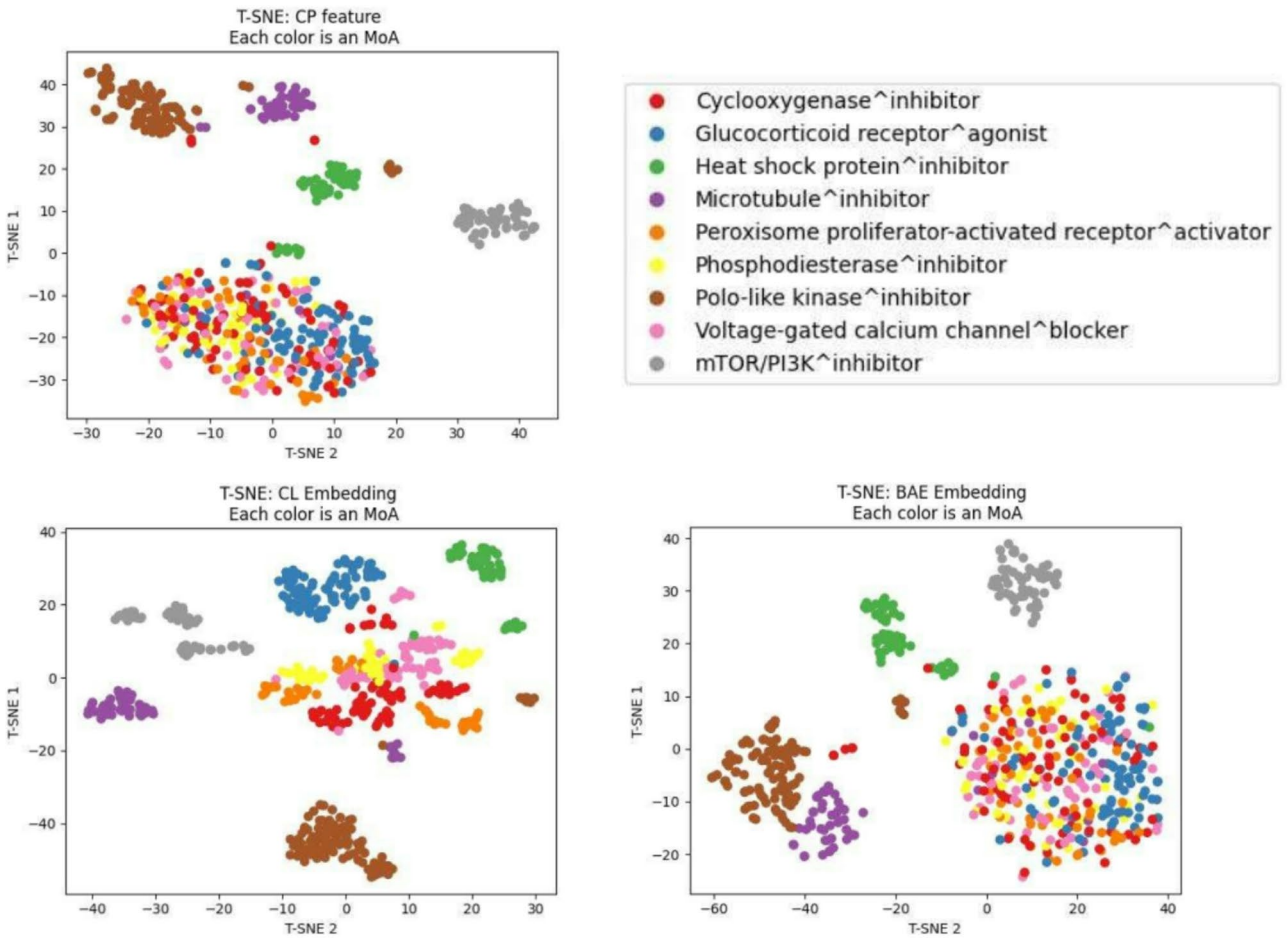
T-SNE plots demonstrating MoA clustering ability of CP feature, CL embeddings and BAE embedding. Visually, CL embeddings greatly improves cluster quality. The improvement is the most pronounced for Glucocorticoid receptor agonist and the Voltage-gated calcium channel blocker clusters.

T-SNE plots were obtained using sklearn.manifold.TSNE from scikit-learn^48^ with parameters (learning rate = ’auto’, init = ’random’, perplexity = 20) (Fig. 4). It could be seen from the CP features and BAE embeddings plots that only four MoA were clearly distinguishable using those features types: Polo-like kinase inhibitor, Microtubule inhibitor, Heat shock protein inhibitor, and mTOR/PI3K inhibitor. The other MoA appeared mixed together. This was not the case for the CL embedding, where we could observe clear clusters formed for those other MoA as well, especially for the Glucocorticoid receptor agonist and the Voltage-gated calcium channel blocker cluster. This was consistent with the kNN accuracy result.

### Bioactivity multitask classification—wider range of tasks

In this section, we revisit the bioactivity multitask comparison, but the features are evaluated on a wider range of tasks. Details about data and model training is the same as in Sect. “Bioactivity multitask classification—tasks that TX performs well but CP does not”). The only difference is that we don’t filter out tasks whose TX features achieved an AUROC > 0.7 and CP features had an AUROC < 0.7. We obtained 703 bioactivity tasks.

Across those tasks, CL embeddings had the highest mean AUROC and RIPtoP-AUPRC (0.687 ± 0.13, 0.343 ± 0.24) among all features types (Table 3), with 294 tasks having AUROC > 0.7 and 164 tasks having AUROC > 0.8. CP features ranked second in terms of mean AUROC (0.680 ± 0.15), mean RIPtoP-AUPRC (0.334 ± 0.25), and number of tasks with AUROC > 0.7 (290). However, CP features had the highest number of tasks with AUROC > 0.8 (169). BAE achieved lower mean scores than both CP features and CL embeddings (0.674 ± 0.14 and 0.325 ± 0.24), with 274 tasks having AUROC > 0.7 and 149 tasks having AUROC > 0.8. TX features had the lowest mean scores overall out of all features types (0.659 ± 0.13 and 0.279 ± 0.22), and lowest number of tasks that had AUROC > 0.7 (252) and AUROC > 0.8 (126). Additionally, we ran a wilcoxon signed rank test for each pair of features type (Supplementary Table 1, 2), with the alternative hypothesis being the row features type outperformed the column features type at significance value 0.05. Overall, CL embeddings and CP features both outperformed BAE embeddings with statistical significance, and all three of them outperformed TX features with statistical significance in terms of both metrics. Despite the higher mean AUROC and RIPtoP-AUPRC, however, the improvement of CL embeddings over CP features was not statistically significant for the Wilcoxon test (p-value 0.0716 and 0.0841, respectively). This is because, overall, CP features outperform CL embeddings in more tasks (354 tasks compared to 349), though in many cases, the difference is minimal (Supplementary Fig. 2), leading to the higher mean scores of CL embedding.

**Table 3:** Performances of each features type for 703 bioactivity classification tasks. Mean metrics ± standard deviation metrics for the mean AUROC and mean RIPtoP-AUPRC columns. # (AUROC > 0.7) denotes number of tasks that achieves AUROC > 0.7.

Features Type	Mean AUROC	Mean RIPtoP-AUPRC	# (AUROC > 0.7)	# (AUROC > 0.8)
CP	0.680 ± 0.15	0.334 ± 0.25	290	169
CL Emb	0.687 ± 0.13	0.343 ± 0.24	294	164
BAE Emb	0.674 ± 0.14	0.325 ± 0.24	274	149
TX	0.659 ± 0.13	0.279 ± 0.22	252	126

To gain deeper insight into which type of biological tasks perform well with which type of features, we investigated the performance of different features types on tasks grouped by five protein target families: G-protein-coupled Transmembrane Receptors, Hydrolase, Ion Channel, Transferase (Kinase), and Cell Proliferation (though not a protein target, it is still a significant assay category). These groups comprised 37, 34, 16, 33, and 50 assays, respectively. We focused on the protein families with the most tasks among the 703 tasks. Of the remaining 553 tasks, 480 were not yet annotated with a protein target family, and 53 were annotated but belonged to protein targets with much fewer tasks.

**Fig. 5:**
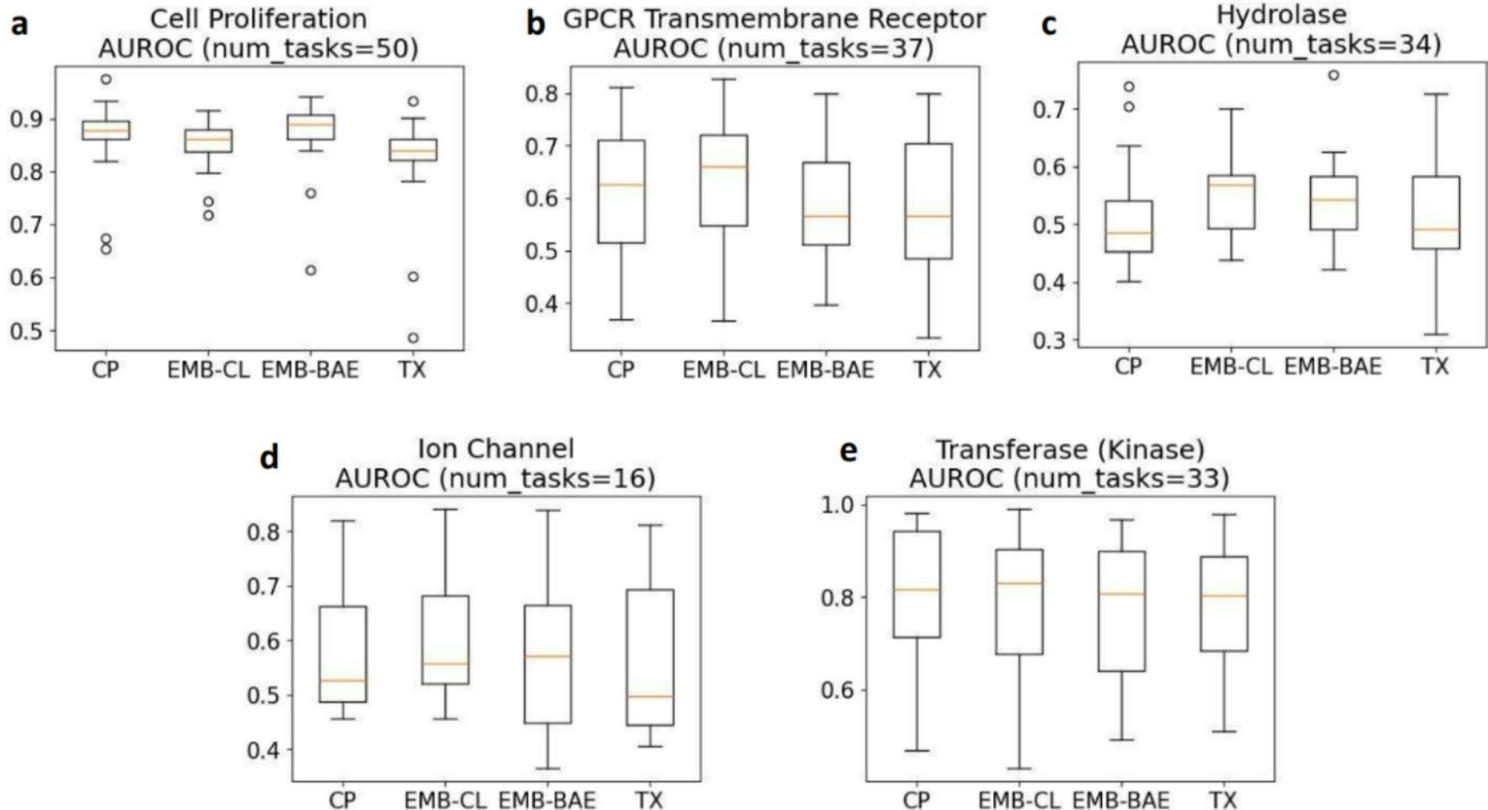
Box plots AUROC for tasks grouped by protein target family. (**a**) Cell Proliferation, (**b**) GPCR Transmembrane Receptor, (**c**) Hydrolase, (**d**) Ion Channel, (**e**) Transferase (Kinase).

**Fig. 6:**
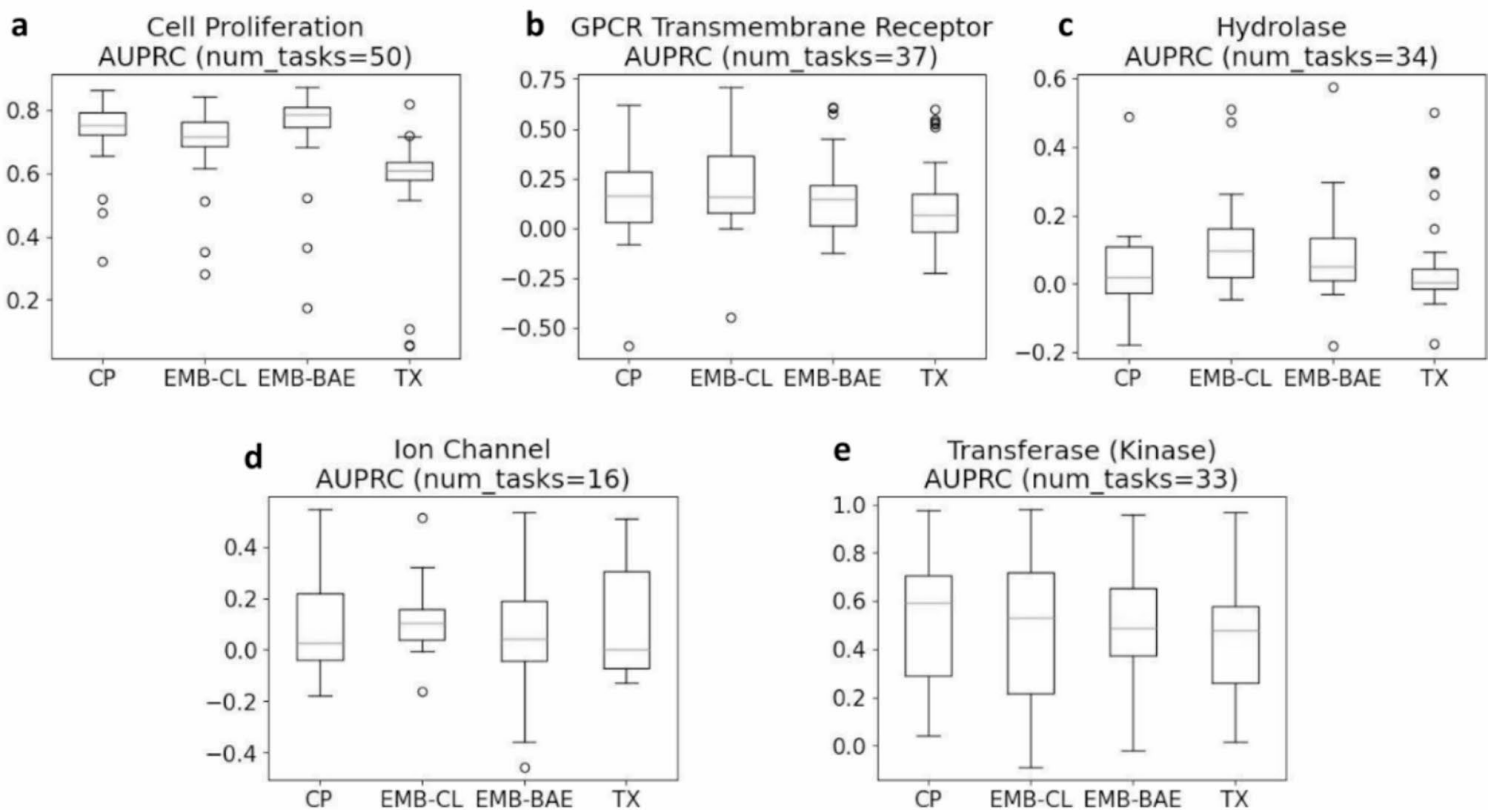
Box plots RIPtoP-AUPRC for tasks grouped by protein target family. (**a**) Cell Proliferation, (**b**) GPCR Transmembrane Receptor, (**c**) Hydrolase, (**d**) Ion Channel, (**e**) Transferase (Kinase).

Overall, for groups of tasks that CP features relatively underperformed, such as GPCR Transmembrane Receptor and Hydrolase tasks, CL embeddings tended to outperform CP features in terms of AUROC (Fig. 5b, c) and RIPtoP-AUPRC (Fig. 6b, c) with statistical significance according to a Wilcoxon signed-rank test at significance level 0.05 (Supplementary Table 5, 6, 7, 8). Ion Channel was also one of such groups, with CL embeddings outperforming CP features in terms of AUROC with statistical significance (Fig. 5d, Supplementary Table 9), but not in terms of RIPtoP-AUPRC where neither features type outperformed another with statistical significance (Fig. 6d, Supplementary Table 10). On the other hand, for groups of tasks that CP features already performed well, for example Transferase (Kinase), CP features tended to outperform both CL and BAE embeddings in terms of AUROC (Fig. 5e), and RIPtoP-AUPRC (Fig. 6e), with statistical significance (Supplementary Table 11, 12). Another group that CP features already performed well on is Cell Proliferation, for which CP features outperformed CL embeddings with statistical significance (Supplementary Table 3, 4) in terms of both metrics (Figs. 5a, 6a). However, it is worth noting that BAE embeddings outperformed both CP features and CL embeddings on Cell Proliferation tasks in terms of both metrics with statistical significance.

## Discussion

We benchmarked two cross-modality representation learning algorithms, contrastive learning (CL) and bimodal autoencoder (BAE), for cell painting (CP) and transcriptomics (TX) data. The aim was to learn a new embeddings from two modalities that could be generated with just one modality (CP). This could address the real-life problem where new compounds would only have CP data, as generating TX data was much more costly. We showed in the supervised bioactivity multitask classification that, in absence of TX features for new compounds due to high cost of data generation, using learned embeddings enhanced performance of CP features on tasks where TX features exceled but CP features does not, with statistical significance from a Wilcoxon signed rank test at significant value 0.05.

Additional investigations showed both visually and through calculating kNN accuracy, that learned representations improved cluster quality for clustering of CP replicates and different mechanisms of action (MoA), demonstrating the learning of biologically meaningful signal. In particular, CL embeddings yielded the best results, while BAE only achieved minor improvements over the CP features. Lastly, returning to the supervised bioactivity multitask classification evaluated on a wider range of biological tasks (703 tasks), we demonstrated that CL embeddings achieved higher mean AUROC and RIPtoP-AUPRC compared to CP features across a range of bioactivity tasks, though the improvements were not statistically significant. Mean scores of BAE embeddings and TX features ranked third and fourth, respectively. However, when tasks are grouped by their protein targets, we observed that CL embeddings outperformed CP features in GPCR transmembrane receptor, hydrolase and ion channel protein families. CP features outperformed both learned embeddings in Transferase tasks, but BAE embeddings outperformed CP features in Cell Proliferation tasks. Overall, for groups of tasks that CP features relatively underperformed compared to TX features, CL embeddings tended to outperform CP features.

## Electronic supplementary material

Below is the link to the electronic supplementary material.

Supplementary Material 1

## Data Availability

The data that support the findings of this study (Cell Painting, RNA-Seq, mechanism of action, and molecule activity data) are proprietary of Johnson & Johnson, and, therefore, cannot be made available. For more information about the data, please contact Steffen Jaensch at sjaensch@its.jnj.com.
