# Supplementary material for: Cross modality learning of cell painting and transcriptomics data improves mechanism of action clustering and bioactivity modelling
Source: Sci Rep. 2025 Jul 2;15:23010. doi: 10.1038/s41598-025-05914-0 (PMC12216865; doi:10.1038/s41598-025-05914-0)
Supplement: Supplementary file 1 — Supplementary Material 1 [file 41598_2025_5914_MOESM1_ESM.docx]

**Supplementary Information**

**Supplementary Table 1: Wilcoxon signed rank tests P-values of the AUROC for 703 bioactivity tasks in Table 2**. The alternative hypothesis is that the AUROC of the row feature being higher than the column feature. P-values smaller than the significant value 0.05 are in bold.

| Feature Type | CP | CL Emb | BAE Emb | TX |
| --- | --- | --- | --- | --- |
| CP | - | 9.28e-01 | **2.73e-04** | **1.02e-10** |
| CL Emb | 7.16e-02 | - | **3.81e-08** | **1.35e-19** |
| BAE Emb | 1 | 1 | - | **5.51e-07** |
| TX | 1 | 1 | 1 | - |

**Supplementary Table 2: Wilcoxon signed rank tests P-values of the RIPtoP-AUPRC for 703 bioactivity tasks in Table 2**. The alternative hypothesis is that the RIPtoP-AUPRC of the row feature being higher than the column feature. P-values smaller than the significant value 0.05 are in bold.

| Feature Type | CP | CL Emb | BAE Emb | TX |
| --- | --- | --- | --- | --- |
| CP | - | 9.16e-01 | **2.83e-03** | **2.59e-22** |
| CL Emb | 8.41e-02 | - | **2.07e-07** | **4.79e-39** |
| BAE Emb | 9.97e-01 | 1 | - | **2.09e-20** |
| TX | 1 | 1 | 1 | - |

**Supplementary Table 3:** **Wilcoxon signed rank tests P-values of the AUROC for Cell Proliferation tasks in Figure 5.** The alternative hypothesis is that the AUROC of the row feature being higher than the column feature. P-values smaller than the significant value 0.05 are in bold.

| Feature Type | CP | CL Emb | BAE Emb | TX |
| --- | --- | --- | --- | --- |
| CP | - | **1.26e-05** | 9.98e-01 | **1.18e-09** |
| CL Emb | 1 | - | 1 | **5.74e-06** |
| BAE Emb | **1.54e-03** | **1.30e-07** | - | **3.58e-09** |
| TX | 1 | 1 | 1 | - |

**Supplementary Table 4: Wilcoxon signed rank tests P-values of the RIPtoP-AUPRC for Cell Proliferation tasks in Figure 6.** The alternative hypothesis is that the RIPtoP-AUPRC of the row feature being higher than the column feature. P-values smaller than the significant value 0.05 are in bold.

| Feature Type | CP | CL Emb | BAE Emb | TX |
| --- | --- | --- | --- | --- |
| CP | - | **2.00e-05** | 1 | **3.78e-10** |
| CL Emb | 1 | - | 1 | **5.44e-10** |
| BAE Emb | **1.45e-04** | **7.47e-07** | - | **4.27e-10** |
| TX | 1 | 1 | 1 | - |

**Supplementary Table 5: Wilcoxon signed rank tests P-values of the AUROC for GPCR Transmembrane Receptor tasks in Figure 5.** The alternative hypothesis is that the AUROC of the row feature being higher than the column feature. P-values smaller than the significant value 0.05 are in bold.

| Feature Type | CP | CL Emb | BAE Emb | TX |
| --- | --- | --- | --- | --- |
| CP | - | 9.63e-01 | **1.12e-02** | **1.53e-02** |
| CL Emb | **3.67e-02** | - | **2.47e-03** | **1.34e-03** |
| BAE Emb | 9.89e-01 | 9.98e-01 | - | **7.14e-02** |
| TX | 9.85e-01 | 9.99e-01 | 9.29e-01 | - |

**Supplementary Table 6: Wilcoxon signed rank tests P-values of the RIPtoP-AUPRC for GPCR Transmembrane Receptor tasks in Figure 6.** The alternative hypothesis is that the RIPtoP-AUPRC of the row feature being higher than the column feature. P-values smaller than the significant value 0.05 are in bold.

| Feature Type | CP | CL Emb | BAE Emb | TX |
| --- | --- | --- | --- | --- |
| CP | - | 9.71e-01 | 2.00e-01 | 6.21e-02 |
| CL Emb | **2.88e-02** | - | **2.88e-02** | **4.41e-03** |
| BAE Emb | 8.00e-01 | 9.71e-01 | - | 3.22e-02 |
| TX | 1 | 1 | 1 | - |

**Supplementary Table 7: Wilcoxon signed rank tests P-values of the AUROC for Hydrolase tasks in Figure 5.** The alternative hypothesis is that the AUROC of the row feature being higher than the column feature. P-values smaller than the significant value 0.05 are in bold.

| Feature Type | CP | CL Emb | BAE Emb | TX |
| --- | --- | --- | --- | --- |
| CP | - | 9.99e-01 | 9.86e-01 | **7.50e-01** |
| CL Emb | **7.83e-04** | - | 2.64e-01 | **8.71e-02** |
| BAE Emb | **1.40e-02** | 7.36e-01 | - | 1.29e-01 |
| TX | 2.50e-01 | 9.13e-01 | 8.71e-01 | - |

**Supplementary Table 8: Wilcoxon signed rank tests P-values of the RIPtoP-AUPRC for Hydrolase tasks in Figure 6.** The alternative hypothesis is that the RIPtoP-AUPRC of the row feature being higher than the column feature. P-values smaller than the significant value 0.05 are in bold.

| Feature Type | CP | CL Emb | BAE Emb | TX |
| --- | --- | --- | --- | --- |
| CP | - | 1 | 9.88e-01 | 4.24e-01 |
| CL Emb | **5.37e-05** | - | 7.93e-02 | **1.08e-02** |
| BAE Emb | **1.15e-02** | 9.21e-01 | - | 6.87e-02 |
| TX | 5.76e-01 | 9.89e-01 | 9.31e-01 | - |

**Supplementary Table 9: Wilcoxon signed rank tests P-values of the AUROC for Ion Channel tasks in Figure 5.** The alternative hypothesis is that the AUROC of the row feature being higher than the column feature. P-values smaller than the significant value 0.05 are in bold.

| Feature Type | CP | CL Emb | BAE Emb | TX |
| --- | --- | --- | --- | --- |
| CP | - | 1 | 3.48e-01 | 3.85e-01 |
| CL Emb | **9.77e-04** | - | 1.88e-01 | 6.54e-02 |
| BAE Emb | 6.88e-01 | 8.39e-01 | - | 6.52e-01 |
| TX | 6.52e-01 | 9.47e-01 | 3.85e-01 | - |

**Supplementary Table 10: Wilcoxon signed rank tests P-values of the RIPtoP-AUPRC for Ion Channel tasks in Figure 6.** The alternative hypothesis is that the RIPtoP-AUPRC of the row feature being higher than the column feature. P-values smaller than the significant value 0.05 are in bold.

| Feature Type | CP | CL Emb | BAE Emb | TX |
| --- | --- | --- | --- | --- |
| CP | - | 8.75e-01 | 3.26e-01 | 4.10e-01 |
| CL Emb | 1.50e-01 | - | 1.80e-01 | 1.02e-01 |
| BAE Emb | 7.15e-01 | 8.50e-01 | - | 6.33e-01 |
| TX | 6.33e-01 | 9.18e-01 | 4.10e-01 | - |

**Supplementary Table 11: Wilcoxon signed rank tests P-values of the AUROC for Transferase (Kinase) tasks in Figure 5.** The alternative hypothesis is that the AUROC of the row feature being higher than the column feature. P-values smaller than the significant value 0.05 are in bold.

| Feature Type | CP | CL Emb | BAE Emb | TX |
| --- | --- | --- | --- | --- |
| CP | - | **1.55e-03** | **8.95e-03** | **3.63e-01** |
| CL Emb | 9.98e-01 | - | 2.81e-01 | 5.25e-01 |
| BAE Emb | 9.91e-01 | 7.19e-01 | - | 5.18e-01 |
| TX | 9.64e-01 | 4.75e-01 | 4.82e-01 | - |

**Supplementary Table 12: Wilcoxon signed rank tests P-values of the RIPtoP-AUPRC for Transferase (Kinase) tasks in Figure 6.** The alternative hypothesis is that the RIPtoPAUPRC of the row feature being higher than the column feature. P-values smaller than the significant value 0.05 are in bold.

| Feature Type | CP | CL Emb | BAE Emb | TX |
| --- | --- | --- | --- | --- |
| CP | - | **2.45e-03** | **1.79e-02** | **3.58e-03** |
| CL Emb | 9.98e-01 | - | 1.25e-01 | 1.36e-01 |
| BAE Emb | 9.82e-01 | 8.75e-01 | - | 2.75e-01 |
| TX | 9.96e-01 | 8.64e-01 | 7.25e-01 | - |

**Supplementary Table 13: Wilcoxon signed rank tests P-values of the AUROC for 47 bioactivity tasks in Table 3.** The alternative hypothesis is that the AUROC of the row feature being higher than the column feature. P-values smaller than the significant value 0.05 are in bold.

| Feature Type | CP | CL Emb | BAE Emb |
| --- | --- | --- | --- |
| CP | - | 1 | 9.62e-01 |
| CL Emb | **4.29e-04** | - | 7.97e-02 |
| BAE Emb | **3.77e-02** | 9.20e-01 | - |

**Supplementary Table 14: Wilcoxon signed rank tests P-values of the RIPtoP-AUPRC for 47 bioactivity tasks in Table 3.** The alternative hypothesis is that the RIPtoP-AUPRC of the row feature being higher than the column feature. P-values smaller than the significant value 0.05 are in bold.

| Feature Type | CP | CL Emb | BAE Emb |
| --- | --- | --- | --- |
| CP | - | 9.99e-01 | 7.58e-01 |
| CL Emb | **1.37e-03** | - | **8.63e-03** |
| BAE Emb | 2.42e-01 | 9.91e-01 | - |


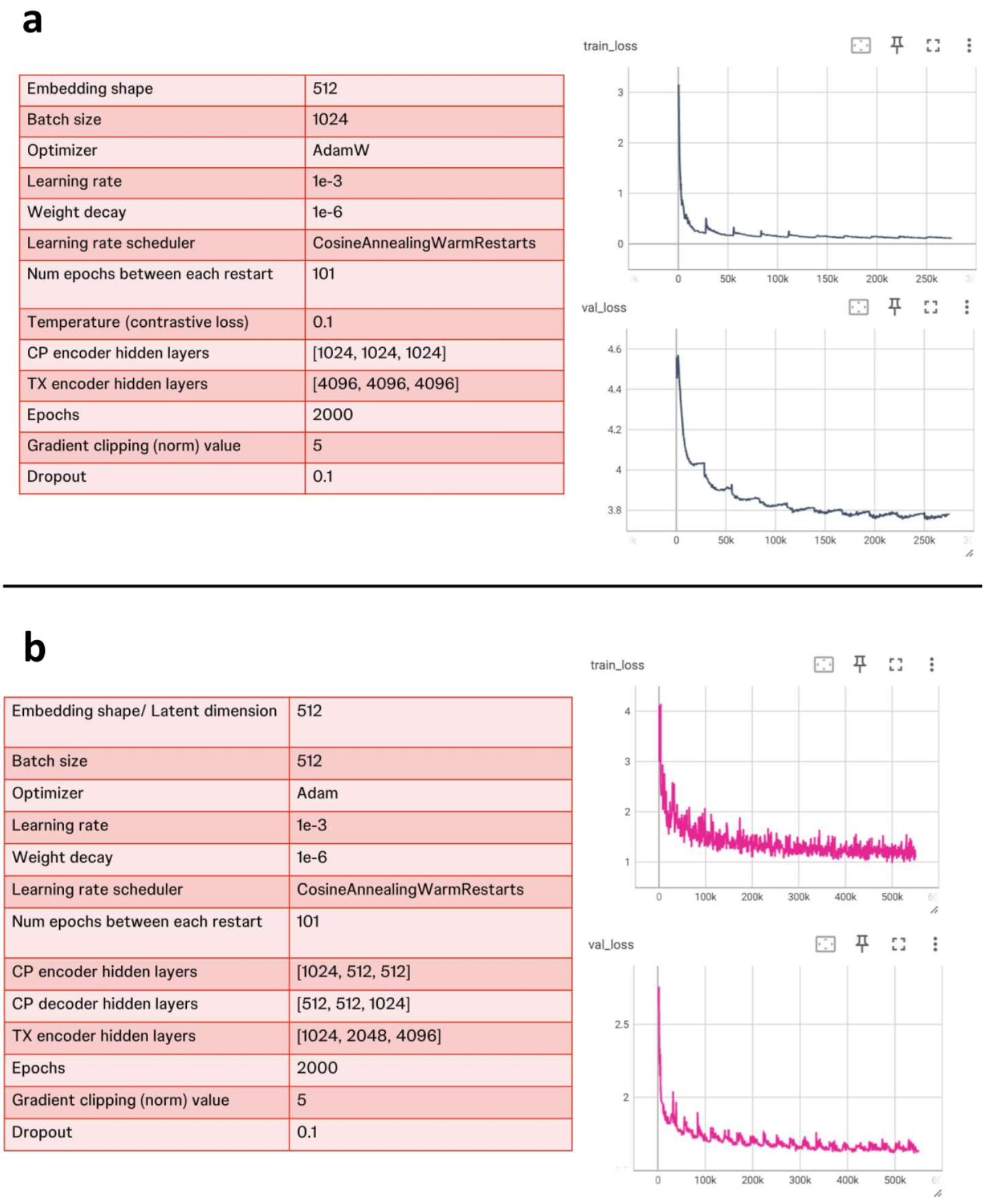


**Supplementary Fig. 1: Hyperparameters and train/validation loss curves for a) contrastive learning, b) bimodal autoencoder.**

**
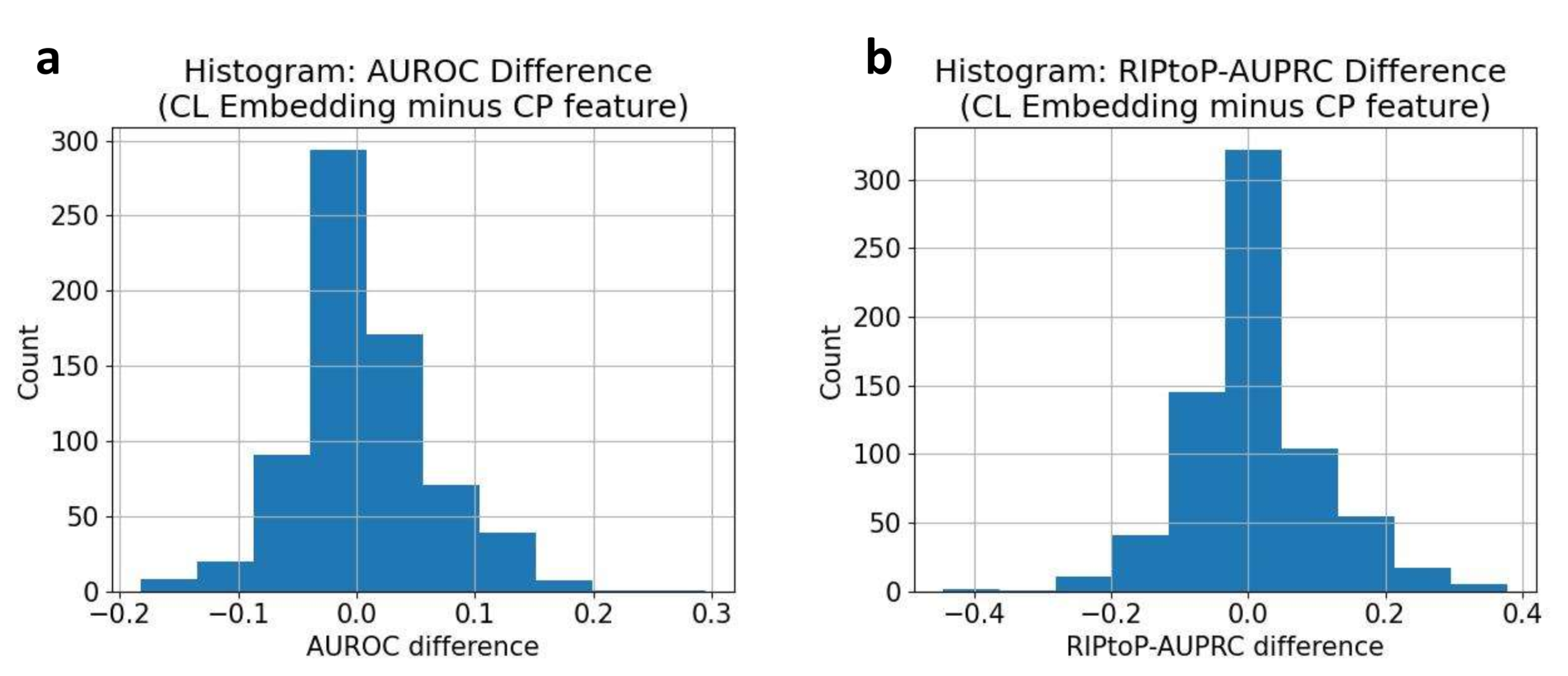
**

**Supplementary Fig. 2: Histogram of a) AUROC b) RIPtoP-AUPRC difference (CL embedding minus CP feature).**

**
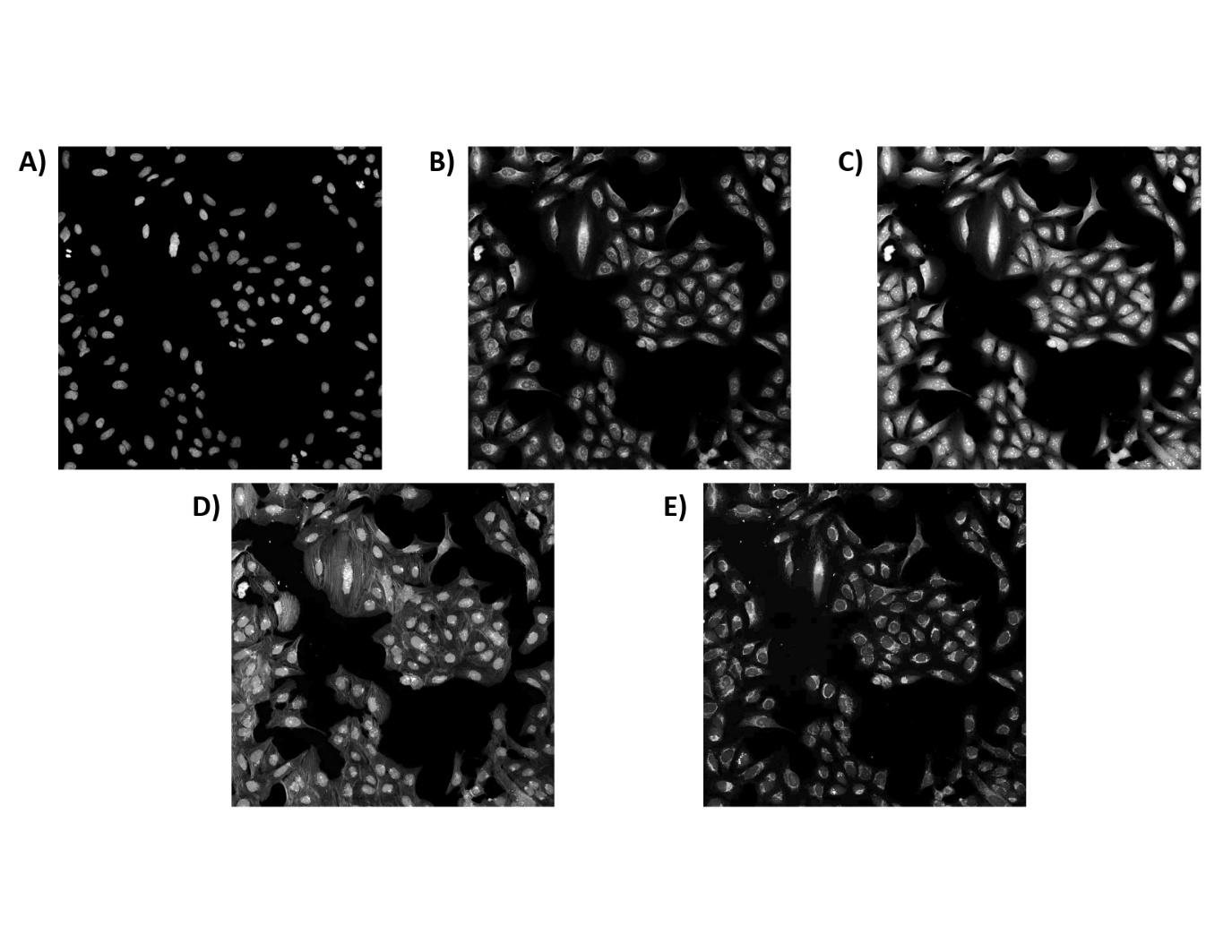
**

**Supplementary Fig. 3: Sample cell painting microscopy images. Five channels of the same view imaged in the Cell Painting protocol. Each highlights a different organelle or cellular component, A) Nucleus, B) Endoplasmic reticulum, C) Nucleoli, cytoplasmic RNA, D) Actin, Golgi, plasma membrane, E) Mitochondria.**
